# Envisioning emerging frontiers on human gut microbiota and its applications

**DOI:** 10.1111/1751-7915.13671

**Published:** 2020-09-24

**Authors:** Marco Ventura, Christian Milani, Francesca Turroni, Douwe van Sinderen

**Affiliations:** ^1^ Laboratory of Probiogenomics, Department of Chemistry, Life Sciences and Environmental Sustainability University of Parma Parma Italy; ^2^ Microbiome Research Hub University of Parma Parma Italy; ^3^ APC Microbiome Institute and School of Microbiology Bioscience Institute National University of Ireland Cork Ireland

## Abstract

The human gut microbiota is involved in multiple health‐influencing host interactions during the host’s entire life span. Microbes colonize the infant gut instantaneously after birth and subsequently the founding and interactive progress of this early gut microbiota is considered to be driven and modulated by different host‐ and microbe‐associated forces. A rising number of studies propose that the composition of the human gut microbiota in the early stages of life impact on the human health conditions at later stages of life. This notion has powered research aimed at detailed investigations of the infant gut microbiota composition. Nevertheless, the molecular mechanisms supporting the gut microbiome functionality and the interaction of the early gut microbes with the human host remain largely unknown.

## Graphical abstract

This manuscript provides a general overview about the future avenues of research in the field of microbiota.

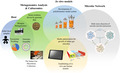

## General features

The human body harbours trillions of microbial cells, which represent thousands of different species whose coordinated actions are presumed to be critical for human health and well‐being (Milani *et al*., 2017). Such microbial cell populations reach their highest density in the gut where they collectively form a complex microbial community known as the gut microbiota (Lozupone *et al*., 2012), which dynamically develops from host infancy to reach a more or less stable and final composition at around three years of age (Yatsunenko *et al*., 2012; Bokulich *et al*., 2016; Yassour *et al*., 2016). Various epidemiological studies have established a clear correlation between (factors that cause) an aberrant gut microbiota composition during childhood on the one hand, and immune and metabolic disorders occurring at later life on the other (Eggesbo *et al*., 2003; Huh *et al*., 2012a; Sevelsted *et al*., 2015a). Thus, there is growing experimental data that support long‐term health benefits elicited by the infant gut microbiota and that also implicate this early human gut microbiota in modulating risk factors related to particular health conditions that manifest themselves during adulthood (Relman, 2012). It has been well documented that bacteria and associated bacteriophages of the gut microbiota of humans and other mammals are vectored from mother to her offspring during delivery by vertical transmission [for a review, see (Milani *et al*., 2017)]. Furthermore, human milk appears to facilitate the acquisition and maintenance of microbes by the newborn after each feeding event. Thus, each feeding event is simultaneously accompanied by the inoculation of the baby by bacteria present in human milk, representing the milk microbiota (Duranti *et al*., 2017; Milani *et al*., 2017). In order to explain the origin of milk microbiota, two routes have been proposed, involving i) entero‐mammary translocation of the maternal gut microbiota and ii) inoculation by the infant’s oral microbiota through regurgitation during breastfeeding (Rodriguez, 2014; Ruiz *et al*., 2019). The occurrence of bacteria in colostrum, when harvested prior the first baby feeding event supports the entero‐mammary route, whereas the observation that pumped breastmilk has a different microbiota composition when compared to that of direct breastfeeding mothers, supports the hypothesis of retrograde inoculation by members the infant oral microbiota (Rodriguez, 2014; Moossavi *et al*., 2019; Ruiz *et al*., 2019).

The intimate association between microbial populations and the human body as their host has led to the formulation of the so‐called holobiont concept. This theoretical concept proposes that a human being (or another animal) acts as a superorganism hosting a plethora of microorganisms that have co‐evolved with their eukaryotic host to produce an intricate network of physical interactions and molecular messages that are crucial to host function and therefore to host health and well‐being (Gilbert, 2014).

Scientific findings have clearly underpinned the key role played by the infant gut microbiota in appropriate host development with its consequences for host health and disease, while it also underscores the exigent need to expand our knowledge on this matter. In fact, a lot of our current knowledge pertaining to the functionalities of the infant gut microbiota is based on *in silico* predictions. For example, particular associations between gut microbiota composition and certain health conditions have been identified, although clear insights into the biology of the implicated gut microbes and the responsible molecular mechanisms are typically lacking. This represents a serious knowledge caveat preventing the definition of reliable predictor parameters of human health and disease based on the resident microbiota. In particular, in many cases, it cannot be ruled out whether a microbiota profile shift in a given patient represents a cause or a consequence of a disease. Such scientific dilemmas expose the need to apply a holistic approach, involving basic microbiology, biochemistry, cell biology, immunology and bioinformatics, which will need to be carried out in order to provide a biological interpretation of the massive amount of *in silico* data sets that have been generated by next‐generation sequencing and associated bioinformatics approaches. Only a multi‐pronged approach involving both *in silico*, laboratory‐based experiments, *in silico* data processing and interpretation, and associated clinical studies will facilitate microbiota‐based translational science, which will move from the paradigm of just describing a phenomenon into a mechanism‐based discipline that will lead to effective (preventative or risk‐reducing) treatments. This will open new avenues of research concerning the understanding of gut microbiome functionality, thereby underpinning the mode of action of microbe–microbe and microbe–host interactions, which are believed to be responsible for various autoimmune diseases and metabolic disorders.

Together, the above underscores the high scientific relevance of understanding the various functional roles played by the gut microbiota during the first stages of life and of identifying host factors that drive the assemblage of the gut microbiota in infants. Nevertheless, despite an ever‐growing enthusiasm for the functional microbiome, advances in gut microbiota functionality and associated molecular mechanisms are still slow and sometimes controversial.

## Culturomics approaches and the rebirth of microbiology

**Fig. 1:**
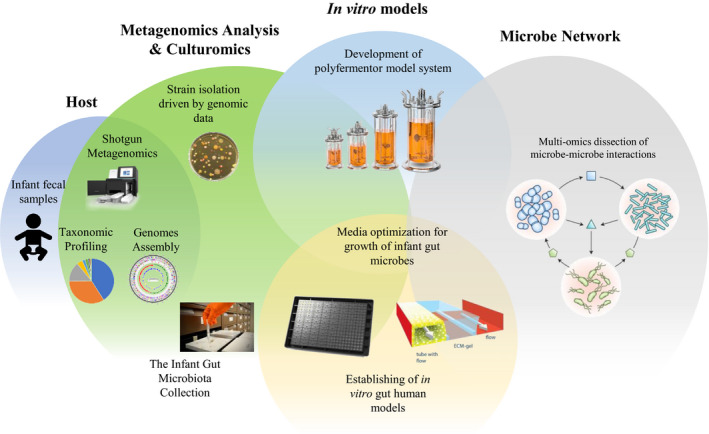
Schematic representations of the *in vitro* models used for underpinning the microbe–microbe and host–microbe cross talks.

As described above the advent of cultivation‐independent approaches, i.e. shut‐gun sequencing and other metagenomic techniques, to assess the biodiversity of complex microbial communities such as that of the human large intestine, have clearly identified the presence of thus far unknown microorganisms. A large number of these microbes appear to be recalcitrant to cultivation and for this reason are considered to constitute the ‘dark matter’ of the gut microbiota (Lagier *et al*., 2018). Concomitantly, environmental microbiology has developed novel approaches aimed at cultivating such unculturable bacteria, largely based on high‐throughput extensions of traditional microbiological media and classical growth protocols, which has prompted the rebirth of microbiological cultivation techniques (Kaeberlein *et al*., 2002). In addition, the use of multiple growth conditions combined with extended incubation times allowed the isolation of several novel bacterial species belonging to the human gut dark matter (Lagier *et al*., 2018). Altogether, these culturing attempts are considered part of the novel ‘omics’ methodology known as culturomics, which involves the application of multiple cultivation conditions for the medium‐to‐high‐throughput isolation of individual bacteria, as well as molecular fingerprinting technologies such as MALDI‐TOF mass spectrometry coupled with 16S rRNA sequencing to support bacterial identification (Lagier *et al*., 2018) (Fig. 1). Thanks to culturomics, we have expanded our view of bacterial biodiversity of the human gut, leading an enhancement of 23 % of the current repertoire of the bacterial taxa to be cultured from human gut samples (Lagier *et al*., 2018). It has been postulated that among the 14 300 known prokaryotic taxa, just 2671 have been isolated at least once from the gut environment (Bollmann *et al*., 2007). Notably, merely 690 prokaryotes species were known before the application of culturomics approaches, and this number has risen to 2671 upon the introduction of these novel microbial cultivation advances (Lagier *et al*., 2018).

The importance of culturomics in the gut microbiota era is exemplified by the fact that such novel omics approaches provide new bacterial strains, which can be used for *in vitro* and *in vivo* experiments to characterize their biological features (e.g. to study metabolic activities and their interaction with the human host). Furthermore, isolation of novel microbial strains by culturomics approaches allows translational applications such as therapeutic agents in bacteriotherapy protocols and novel antimicrobial agents (e.g. bacteriocins and other antimicrobial peptides). Nevertheless, one of the main shortcomings of culturomics is represented by the substantial human resource requirement and the impossibility to test as many samples as other methods like metagenomics. Nevertheless, recent technological advents, e.g. the development of sophisticated (semi‐)automated systems based on robots are promising in terms of rendering the culturomics approaches high‐throughput and thus capable of processing thousands of samples timely. Another major limitation of culturomics is that it cannot identify so‐called ‘not yet culturable’ microorganisms (Lagier *et al*., 2018), i.e. those microorganisms that were identified by metagenomics analyses but that have yet to be isolated. However, in order to overcome this drawback, a ‘hybrid approach’ has recently been developed, involving both shotgun metagenomic analyses and culturomics attempts, which allowed the isolation of ‘not yet culturable’ bifidobacterial species (Lugli *et al*., 2019). This approach employs metagenomics‐based metabolic modelling to identify nutritional requirements of bacterial taxa that have been identified as novel by shotgun metagenomic analyses, thereby allowing the formulation of culturomics protocols for their cultivation and subsequent genetic characterization (Lugli *et al*., 2019).

Functional characterization of (elements of) the gut microbiome, including that associated with early human life, is still marred with major limitations, in particular the lack of comprehensive public repositories of bacterial strains, which have been isolated from the human gut and which a given laboratory can have access to perform dedicated experiments. In fact, assuming that one of the new frontiers of gut microbiota science is to perform efforts to cultivate and characterize gut commensals that fully represent the genetic diversity highlighted by metagenomics analyses, another crucial aim will be to archive these gut bacteria in a pure culture and viable form in well‐organized, publicly accessible bacterial culture collections.

## The need to investigate host–microbe interactions in the human gut

During the last ten years the human gut microbiota has enjoyed astonishing progress in terms of the generation of relevant metagenomics data sets. In contrast, the understanding of functional features of the human gut microbiome still lags behind, remaining at a relatively immature level as a consequence of its complexity represented by thousands of members that are presumed to engage in a molecular dialogue with the human host as well as with each other. Such interactions may explain the functional role/s played by the gut microbiota in terms of modulating human health and well‐being. Thus, there is an obvious need to investigate and understand the intricate networks of interactions occurring between the human host and gut microbes. These microbe–host phenomena are particularly important during the first stages of life when assembly of the gut microbiota is still in progress and the physiology, gut metabolism and immune system of the host is still immature and therefore prone to influences that may be directed by specific microbiota elements. To address this crucial research gap, there is a need for a reductionist approach in which both host and microbiome are streamlined to the level that experimental variables can be tightly controlled and easily modified (Fig. 1). In this context, the human gut organoids, which are implemented as the ‘gut‐on‐a‐chip’ model, and which are self‐organizing three dimensional epithelial structures originated from stem cells are recent developments employed to simulate a host (Kim *et al*., 2012; Shah *et al*., 2016; Kasendra *et al*., 2018). The ‘gut‐on‐a‐chip’ represents a microfluidic device in which cells are cultured with organ‐relevant spatiotemporal chemical gradients and dynamic mechanical cues, thus directed to reconstruct the structural tissue arrangements and functional complexity of a living organ in an *in vitro* setting (Huh *et al*., 2012b). Remarkably, unlike other *in vitro* host models such as human cell monolayers, such a device represents a dynamic model that mimics gut peristalsis, while it includes endothelial cells and peripheral blood mononuclear cells, and as such is believed to provides a realistic reflection of the human gut ecosystem.

Currently available studies focusing on the understanding of host–microbe interactions are predominantly based on murine models (conventional as well as axenic mice; Hugenholtz and de Vos, 2018). However, mice and other animal models do come with serious limitations that preclude direct translation of research results to humans (Nguyen *et al*., 2015).

With respect to the development of appropriate *in vitro* model simulating the human gut microbiota, synthetic or defined communities have been successfully employed as gut microbiota models. Most of these were achieved due to sophisticated culturomics approaches, that allow the establishment of defined microbial communities, known as synthetic bacteriomes, which are evocative of the adult human gut microbiota [for a review see (Elzinga *et al*., 2019)]. These synthetic bacteriomes are based on the concept of ‘a core gut microbiota’, i.e. a conserved set of microbial taxa in the human gut, reflecting the subdivision of the gut microbiota in enterotypes (Arumugam *et al*., 2011). This core gut microbiota encompasses keystone species, whose roles are fundamental to the ecosystem structure and function (Trosvik and de Muinck, 2015). Comparing the core gut microbiota with the microbiota composition found in various diseases facilitates the identification and subsequent design of synthetic bacteriomes representative of a healthy or disease‐associated gut microbiota. In the quest for knowledge on microbe–microbe interactions it will be imperative to have access to *in vitro* models in which synthetic bacteriomes can be applied. In this context, the use of fermentation models has proven successful in shaping the gut microbiota under *in vitro* conditions, ranging from short‐term batch incubations to multi‐compartmental continuous cultivation systems (Payne *et al*., 2012; Venema and van den Abbeele, 2013). However, most of these models do not include all host components, except for certain environmental parameters that reflect parts of the gut (e.g. pH, bile acids/salts and/or mucin) and that can be added to the model and/or modified. These shortcomings limit the value of these models in terms of their relevance to host–microbe interactions, though they may still be important to assess microbe–microbe interactions.

## Conclusions

In the last decade, very substantial efforts have been made to dissect and characterize the infant gut microbiota composition using metagenomics approaches. However, comparatively few studies have been dedicated to disentangle the early life microbiota interactions between microbes and their mammalian host, and to dissect how their composition and associated metabolic activities impact on human health. In this context, there are fundamental and commercial interests aimed at utilizing microbiota members and/or their metabolites as reliable predictors for human disease risk, and at developing rational strategies, e.g. based on prebiotics and or novel probiotics, to modulate early life gut microbiota to reduce or eliminate disease risks, such as those relevant to allergies, chronic immune disorders and metabolic disorders, in adulthood (Eggesbo *et al*., 2003; Sevelsted *et al*., 2015b). Such health risks are higher in babies born by caesarean section (CS) compared to those born naturally, apparently due to the lack of acquisition of a gut microbiota through vertical transmission of microbes from their mother (Wampach *et al*., 2018). Thus, future preventative clinical practices in paediatric medicine may be directed to promote colonization of CS‐delivered babies with a synthetic bacteriome composed of infant gut commensals, known to be commonly acquired from the mother during natural delivery, and able to interact with the host in order to reduce/abolish risks of development of later life diseases.

Nonetheless, these intervention approaches require an in depth assessment of the composition of the human early life gut microbiota and identification of crucial microbiota elements that play a role in the aetiology of diseases. Another crucial window of opportunity that could be targeted in order to modulate the assembly of the first gut microbiota in human is pregnancy. Manipulation of the gut microbiota of the mothers during pregnancy is postulated to modify the assemblage of the infant gut microbial communities in early life. However, many unanswered questions remain concerning how and why certain bacteria are specifically acquired by the baby as a result of vertically transmission from their corresponding mother. In addition, we currently do not know anything about the persistence (or residency) of specific bacteria in the gut of the baby following weaning, and about their impact on host heath. We expect that, given that the discovery of the complexity of the human gut microbiota has revolutionized life sciences in the most recent decade, in the near future the microbiota discipline will transform human life in terms of its impact on health and well‐being.

## Conflict of interest

None declared.
